# Unique protein features of SARS-CoV-2 relative to other Sarbecoviruses

**DOI:** 10.1093/ve/veab067

**Published:** 2021-07-23

**Authors:** Matthew Cotten, David L Robertson, My V T Phan

**Affiliations:** MRC/UVRI & LSHTM Uganda Research Unit, Plot 51-59 Nakiwogo Road, P.O Box 49, Entebbe, Uganda; MRC-University of Glasgow Centre for Virus Research, Sir Michael Stoker Building, Garscube Campus, 464 Bearsden Road, Glasgow G61 1QH, UK; MRC-University of Glasgow Centre for Virus Research, Sir Michael Stoker Building, Garscube Campus, 464 Bearsden Road, Glasgow G61 1QH, UK; MRC/UVRI & LSHTM Uganda Research Unit, Plot 51-59 Nakiwogo Road, P.O Box 49, Entebbe, Uganda

**Keywords:** SARS-CoV-2, proteome changes, *Sarbecovirus* evolution, spike protein changes

## Abstract

Defining the unique properties of severe acute respiratory syndrome coronavirus 2 (SARS-CoV-2) protein sequences has potential to explain the range of Coronavirus Disease 2019 severity. To achieve this we compared proteins encoded by all Sarbecoviruses using profile Hidden Markov Model similarities to identify protein features unique to SARS-CoV-2. Consistent with previous reports, a small set of bat- and pangolin-derived Sarbecoviruses show the greatest similarity to SARS-CoV-2 but are unlikely to be the direct source of SARS-CoV-2. Three proteins (nsp3, spike, and orf9) showed regions differing between the bat Sarbecoviruses and SARS-CoV-2 and indicate virus protein features that might have evolved to support human infection and/or transmission. Spike analysis identified all regions of the protein that have tolerated change and revealed that the current SARS-CoV-2 variants of concern have sampled only a fraction (∼31 per cent) of the possible spike domain changes which have occurred historically in *Sarbecovirus* evolution. This result emphasises the evolvability of these coronaviruses and the potential for further change in virus replication and transmission properties over the coming years.

## 1. Introduction

Since the first report of Coronavirus Disease 2019 (COVID-19) caused by severe acute respiratory syndrome coronavirus 2 (SARS-CoV-2) in December 2019 in Wuhan city, China (Li et al., 2020; Yang et al., 2020) and the declaration of COVID-19 a global pandemic in March 2020 by the World Health Organization, the disease has proceeded to affect every part of the world. The SARS-CoV-2 virus belongs to the *Coronaviridae* family of enveloped positive-sense single-stranded RNA viruses, *Betacoronavirus* genus, *Sarbecovirus* subgenus. Other Sarbecoviruses include SARS-CoV (the coronavirus causing the SARS outbreak in 2002–2004) and a large number of SARS-like bat viruses. The genomes of Sarbecoviruses are 30 kb in length, encoding >14 open reading frames (ORFs). Among the structural proteins, the spike protein plays a crucial role in virus host-cell tropism, host range, cell entry, and infectivity and is considered the main protein target for protective immune responses. Other virus ORFs encode structural and accessory proteins, many of which modulate important host responses to infection.

Investigation of the evolutionary history of SARS-CoV-2 shows a clear link to Sarbecoviruses circulating in horseshoe bats although no direct animal precursor for SARS-CoV-2 has been identified (Andersen et al., 2020; Boni et al., 2020; Zhang, Wu, and Zhang 2020; Zhou et al., 2020a; Lam et al., 2020). We sought to identify unique peptide regions of SARS-CoV-2 compared to all available Sarbecoviruses to determine viral features that might be unique to SARS-CoV-2 and that might have allowed the virus to infect, replicate, and transmit efficiently in humans. Such a comparative analysis of viral proteins might provide insights into the origin of the virus and identify the conditions that led to the zoonosis to humans, efficient spread without the need for much, if any, adaptation (MacLean et al., 2021), as well as provide leads for drug and immune targets for effective treatments.

## 2. Results and discussion

### 2.1 Protein domains and profile hidden Markov models

We have explored the genomes across the *Sarbecovirus* subgenus using profile hidden Markov models (pHMMs). pHMMs can provide a detailed statistical description of an amino acid (aa) sequence and can be used to detect related domains found and to document their differences from a reference domain (Eddy 1998, 1996). Efficient tools for preparing and comparing pHMMs are available in the HMMER-3 package (Eddy 2011). This approach is particularly useful for comparing large or evolutionary divergent genomes. We have recently used these methods to identify and classify diverse coronaviruses in the *Coronaviridae* family (Phan et al., 2018) and to explore large and unwieldy genomes such as those from the African Swine Fever Virus (Masembe et al., 2020). Here pHMMs were used to explore the relationship between SARS-CoV-2 and the other known Sarbecoviruses to gain understanding of their evolutionary history and to identify regions of encoded viral proteins that are static to change or are altered across the Sarbecoviruses.

### 2.2 Genome scans using custom pHMM domains

**Figure 1.**
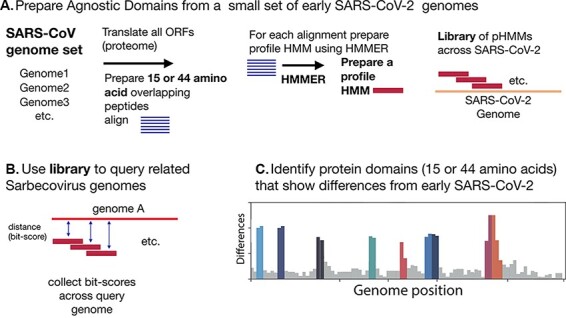
Analysis scheme. (A) pHMM 44 aa domains were generated from a set of 35 early (Pango) lineage B SARS-CoV-2 genome sequences. All ORFs were translated and then sliced into either 44-aa peptides with a step size of 22 aa or 15-aa peptides with a step size of 8 aa. The peptides were clustered using Uclust (Edgar 2010), aligned with MAFFT (Katoh and Standley 2013) and then each alignment was built into a pHMM using HMMER-3 (see Supplementary Material Methods). (B) The set of pHMMs was used to query *Sarbecovirus* genome sequences; bit-scores were collected as a measure of similarity between each pHMM and the query sequence. (B) Bit-scores were gathered and analysed to detect regions that differ between early SARS-CoV-2 genomes and query genomes.

First, some background on the strategy used here is provided. We sought to define the distance of any query virus genome from the early SARS-CoV-2 genome that first began to infect humans in December 2019. To give two levels of resolution, we generated pHMMs from overlapping 44- or 15-aa peptides from all early lineage B SARS-CoV-2 (see Fig. 1A and Supplementary Material Methods). The resulting libraries of pHMMs were then used to survey domain diversity across query coronaviruses relative to the initial 2019 SARS-CoV-2. For each pHMM match to a related sequence, a bit-score is generated, which provides a metric for how close the query sequence is to the pHMM (Fig. 1B). These bit-scores, when collected across an entire viral genome, can provide a sensitive description of similarities and differences between a query genome and the reference genome (Fig. 1C). For additional background on the method, Supplementary Fig. S1 demonstrates the sensitivity of pHMMs to detect and distinguish single aa substitutions and Supplementary Fig. S2 demonstrates the use of pHMMs to identify single aa substitution in a crucial region of the SARS-CoV-2 spike protein.

**Figure 2.**
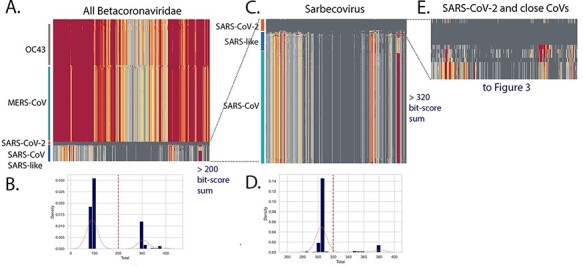
Triage of Betacoronaviruses. All *Betacoronavirus* genomes (excluding SARS-CoV-2) were retrieved from GenBank using the query ((txid694002[Organism] AND 24,000[SLEN]:40,000[SLEN] NOT patent)) NOT txid2697049[Organism]) generating a set of 1,581 genomes that were screened to remove genomes with gaps to yield a set of 1,480 genomes. A small set of 27 early lineage B SARS-CoV-2 genomes from December 2019/January 2020 were added as markers. A library of 44 aa pHMMs prepared from early B SARS-CoV-2 genomes was used and the bit-scores for each pHMM were gathered and used to cluster the genomes (A) with each domain score indicated by colour (dark grey = 1 = very similar to SARS-CoV-2 to red = low = distant from SARS-CoV-2). The total bit-scores sum for each genome was calculated (see histograms of all total bit-score sums (B)). A total bit-score sum of 200 was used to select for the CoV genomes most similar to SARS-CoV-2. The clustering of this subset of CoV genomes (C) included the SARS-CoV-2 genomes, a large number of SARS-CoV genomes, and a smaller number of SARS-like CoVs. A cut-off of 320 for total bit-scores sum (D) was used to identify the closest CoV genomes which were then used for the subsequent analyses reported in Figs. 3, 4, and 5.

An initial triage was performed using all available Betacoronavirus genomes from GenBank on 26 April 2021. All full genomes (length >29,000 nt) with the taxon id 694,002 were retrieved, and genomes with gaps were removed to yield a set of 1,480 Betacoronavirus genomes. SARS-CoV-2 genomes were initially excluded from the retrieval and then a set of 27 early lineage B genomes were added as a reference. An additional five recently reported bat CoV genomes from GISAID were also added (see Supplemental Table S1). The 44 aa pHMM library was used to query the Betacoronavirus set. For each genome, the bit-score for each of 384 pHMMs from early lineage B SARS-CoV-2 sequences was collected and hierarchical clustering based on the normalised domain bit-scores was performed (Fig. 2A). Scores coloured with dark to light grey indicating domains identical or close to the corresponding domain from early lineage B SARS-CoV-2 and yellow to orange to red indicating increasing distance. Within the Betacoronavirus set, the genomes clustered by their taxonomic group and clusters of OC43, MERS-CoV and SARS-CoV and SARS-CoV-2 were observed. The central region of the *Sarbecovirus* genome is conserved across the genome set with all domains marked as dark or light grey in Fig. 2A. This is not unexpected as this central region encodes the viral polymerase, other enzymes, and non-surface-exposed structural proteins of the virus, which are functionally constrained and less likely to allow change than other regions of the virus. In contrast, the domains displayed in yellow, orange, and red in Fig. 2A indicated more increasingly divergent regions between early SARS-CoV-2 and the query *Sarbecovirus* genomes (much lower normalised bit-scores).

The sum of the entire set of bit-scores for a genome was then used to calculate a distance from the early SARS-CoV-2 genome. A histogram of these bit-score sums show several peaks (Fig. 2B) with majority of the Betacoronavirus genomes (mostly OC43 and MERS-CoV) clustering around 100 units and a subset of virus genomes with bit-scores >200 units. This >200 set included the SARS-CoV-2, SARS-like CoVs from bats, and all the SARS-CoV genomes (Fig. 2C). A second triage retained a set of close genomes all with bit-score sums >320 (Fig. 2E) that was used for more detailed analysis. For simplicity, the 27 early B SARS-CoV-2 genomes in the set (which were nearly identical) were reduced to 5, resulting in 19 genomes in the close set: 14 bat/pangolin CoV and 5 SARS-CoV-2 (Fig. 2E).

**Figure 3.**
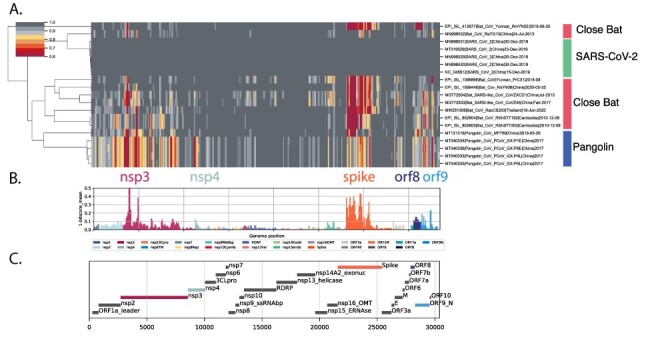
Proteome differences in SARS-CoV-2 versus close bat Sarbecoviruses. All forward ORFs from the 35 early lineage B SARS-CoV-2 genomes were translated, processed into 44-aa peptides (with 22-aa overlap), clustered at 0.65 identity using Uclust (11), aligned with MAAFT (12), and converted into pHMMs using HMMER-3 (Eddy 2011). The presence of these domains was sought in a set of *Sarbecovirus* genomes plus the SARS-CoV-2 genomes. These were then clustered using hierarchical clustering based on the normalised domain bit-scores (e.g. the similarity of the identified query domain to the reference lineage B SARS-CoV-2 domain). Each row represents a genome, and each column represents a domain. Domains are displayed in their order across the SARS-CoV-2 genome, Red = low normalised domain bit-score (lower similarity to lineage B SARS-CoV-2), i.e. higher distance from lineage B SARS-CoV-2, darkest grey = normalised domain bit-score = 1, i.e. highly similar to lineage B SARS-CoV-2. Groups of coronaviruses are indicated to the right of the figure. (A) Domain differences across the *Sarbecovirus* subgenus. (B) For each domain the mean bit-score was calculated across the entire set of *Sarbecovirus* genomes and the value 1-mean bit-score was plotted for each domain. Domains are coloured by the proteins from which they were derived with the colour code indicated below the figure. (C) Schematic of ORFs or protein products of SARS-CoV-2.

We next focused on the bat Sarbecoviruses with the closest similarity to SARS-CoV-2 in at least part of their genomes due to recombinant histories (see Supplementary Table S1 for genome details and references). The clustermap and variance analysis (Fig. 3A) showed higher similarity across most of the genome (dark grey sectors) with three proteins (nsp3, spike, and orf9) displaying reduced bit-scores compared to SARS-CoV-2 (Fig. 3A, yellow and red domains). These differing regions between the bat Sarbecoviruses and SARS-CoV-2 indicate virus protein features that might have evolved to support human infection and/or transmission. The spike differences are explored in detail below; however, it may be important to consider nsp3 and ORF9 (and perhaps nsp4 and ORF8) in future analyses.

### 2.3 Spike changes with 15 aa domains

**Figure 4.**
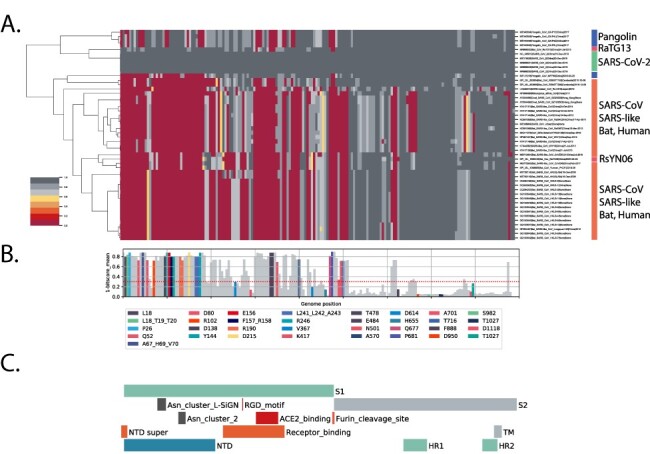
Spike differences in SARS-CoV-2 versus close bat Sarbecoviruses. All forward spike ORFs from the 35 early lineage B SARS-CoV-2 genomes were translated, processed into 15-aa peptides (with 8-aa overlap) and processed into an pHMM library as described in Fig. 2. (A) Shows a hierarchical clustering of 15-aa domain bit-scores. (B) Shows the 1-mean of each domain bit-scores across the genome set, domain values, individual domains that span known aa changes in the 6 VOC and VOIs (B.1.1.7 (Alpha), B.1.351 (Beta), B.1.525 (Eta), P.1 (Gamma), B.1.617.2 (Delta), and A.23.1) are coloured (see key below Panel B). (C) The locations of important spike protein features are indicated. NTD: N-terminal domain, RBD: receptor-binding domain, S1: Spike 1, S1: Spike 2, TM: transmembrane domain, HR1: helical repeat 1, HR2: helical repeat 2, NTD super: N-terminal domain supersite.

Using the same strategy described in Fig. 3, we performed a triage of the Betacoronaviruses with 15 aa pHMMs prepared from early lineage B spike protein (Fig. 4) and selected for CoV genomes encoding close spike proteins. The high bit-score spike set largely overlapped with the high bit-score full genome set suggesting that spike is a good surrogate for full genome homology.

Key features of the spike protein are outlined in Fig. 4C. The analysis revealed regions of spike that historically have tolerated change. In general the S1 subunit of spike (the amino-terminal half of the protein) displayed a large amount of diversity with most of the low score domains (more distant from SARS-CoV-2, marked in red) concentrated here (Fig. 4A). This is consistent with the surface exposure of S1 on the virion and with protein changes driven by pressure to avoid immune responses. The central angiotensin converting enzyme 2 receptor-binding region (Fig. 4C) was very different between the close Sarbecoviruses and SARS-CoV-2. The furin cleavage site at the junction between the S1 and S2 domains (Fig. 4C) is also a region showing a lot of diversity across the *Sarbecovirus* spikes (Fig. 4A) and appears completely unique to SARS-CoV-2. This has been discussed in detail (Hoffmann, Kleine-Weber, and Pöhlmann 2020) and is also a site of frequent change in the current SARS-CoV-2 Variant of Concern (VOC) spike sequences with Q677, P681, and T717 flanking the furin site showing changes (Fig. 4C).

The domains where aa changes have appeared in VOC spike proteins are marked in colour (Fig. 4B) and largely appear in domains with high variation (Fig. 4B, 1-mean bit-score > 0.3) suggesting that Sarbecoviruses have made changes in these regions in previous evolutionary periods and are continuing to change in SARS-CoV-2 evolution. Of the 88 spike domains showing high variation in Sarbecoviruses (1—mean bit-scores ≥ 0.3 units), only 27 of the domains (31 per cent) have accumulated substitutions or deletions. This indicates a very large potential in the SARS-CoV-2 spike protein for tolerating future change. Important regions that have shown high levels of historical change are the NTD, the RBD, and the furin cleavage site and flanking regions.

### 2.4 Global proteome similarities

As described in Fig. 2, a measure of the total protein distance between the SARS-CoV-2 and any query *Sarbecovirus* can be obtained by summing the normalised bit-scores (SNBSs) across the entire query proteome. We examined SNBSs grouped by virus host for the 44 aa total genome analysis and the 15 aa spike gene analysis. The potential role of pangolins as an amplifying intermediate host of SARS-CoV-2 is important to document securely, to guide efforts to prevent or prepare for future zoonotic events. A small number of Sarbecoviruses have been identified in samples from trafficked pangolins in China (Liu, Chen, and Chen 2019; Lam et al., 2020; Xiao et al., 2020), yet there is no direct evidence that pangolins host the virus in their natural environment. It is thus likely that these pangolins identified in China were infected by viruses encountered after transport to China, consistent with reports of disease in these animals. Five CoV sequences from pangolins were included in this analysis (Supplementary Table S1), including four generated by Lam et al., (2020) after sequencing the original samples described by Liu, Chen, and Chen (2019); a 5th genome (MP789) was deposited by Liu et al.

**Figure 5.**
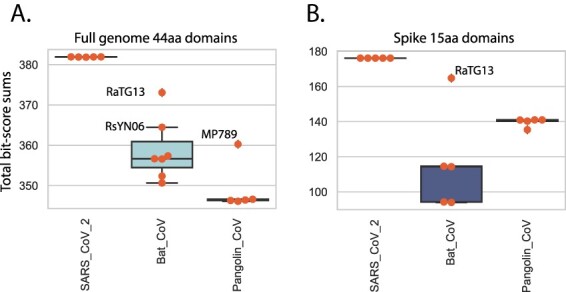
Total domain distances between virus groups. NBSS grouped into SARS-CoV-2 and Sarbecoviruses from pangolin and bat for all domains for each genome were summed. The boxplot shows individual values marked in orange, median values are indicated by horizontal black lines, and first interquartile ranges are marked with a box. The identities of several high scoring bat and pangolin genomes are indicated. (A) NBSS for 44-aa domains across the entire coronavirus genome. (B) NBSS for 15-aa domains across the spike protein.

The bat coronavirus genome RaTG13 (GenBank MN996532.1) was identified as closely related to the SARS-CoV-2 lineage (Zhou et al., 2020b) and supports a bat coronavirus being the zoonotic source of the epidemic, although despite the close genetic distance it is too far in time (decades) for RaTG13 itself to be a direct source of the pandemic SARS-CoV-2 (Boni et al., 2020). The next closest bat coronavirus RsYN06 shows some regions of even close identity to SARS-CoV-2 than RaTG13 (Zhou et al., 2020a) (Fig. 4A) due its possible recombinant nature. A single pangolin-derived SARS-CoV-2 (MP789) showed an SNBS value that was also elevated but not as high as the RaTG13 (Fig. 5A), the 15 aa spike analysis showed similar patterns except that only the RaTG13 spike displayed a high similarity to SARS-CoV-2 (Fig. 5B).

## 3. Conclusions

What is special about SARS-CoV-2? Spike changes in SARS-CoV-2 compared to the close set of *Sarbecovirus* genomes indicate that the immediate zoonotic source of SARS-CoV-2 is yet to be identified due to the unique nature of the SARS-CoV-2 genome. The more detailed analysis of spike regions in SARS-CoV-2 genomes (Fig. 3) revealed the extent of the changes that have occurred across the Sarbecoviruses. Combined with the current VOC spike changes (from lineages B.1.1.7, B.1.351, B.1.525, P.1, B.1.617.2, and A.23.1), the patterns suggest that SARS-CoV-2 has a great deal of possibilities for further evolution, presumably enabling persistence and avoiding immune responses. This emphasises the importance of genomic variant surveillance for monitoring further changes in virus biology that may have implications for spread and disease severity. Vaccine producers should be prepared to accommodate such spike changes in the next generation of vaccine updates. In addition to the spike protein, additional regions of high variance were observed in the nsp3 across all Sarbecoviruses (Fig. 2) in close bat and pangolins (Fig. 3).

The high variance regions flanked and partially overlapped the macro domain, which is frequently associated with ADP-deribosylase activity (Frick et al., 2020; Lei, Kusov, and Hilgenfeld 2018). Variance observed in the ORF8 changes across the set due to frequent deletion of this ORF, suggesting that the encoded protein may be dispensable for human infection. Similar loss of ORF8 was observed with the original SARS-CoV (Chiu et al., 2005; Tang et al., 2006) and has been observed in several SARS-CoV-2 lineages as the virus adapted to humans (Su et al., 2020; Gong et al., 2020; Young et al., 2020). The ORF9 (N protein) variance observed across Sarbecoviruses and the changes in this protein in VOC strains suggest an additional region that may be adapting to human replication. The regions of variance identified here may indicate either functional changes in SARS-CoV-2 proteins or aa positions that can be changed without impairing the necessary functions of the protein. The relatively high mutation rate of SARS-CoV-2 combined with the unprecedented number of SARS-CoV-2 infections in the world is resulting in massive viral adaptation. Additional experiments are required to distinguish true functional changes from neutral evolution.

Finally, and most importantly, the detailed spike analysis of Fig. 4 revealed 88 15-aa spike domains showing high variation while only 27 of these (31 per cent) have accumulated substitutions or deletions in the current epidemic VOC and VOI genomes indicating a large potential for tolerating future change. It is highly likely that new SARS-CoV-2 variants with changes in these regions will evolve, compatible with similar levels of virus replication but tolerating significant antigenic change in the coming years, unless global SARS-CoV-2 spread is severely curtailed.

## Data Availability

All sequence data used in this manuscript are from public databases and sources are cited in the Supplementary data Table 1.

## Supplementary Material

veab067_Supp
