# Supplementary material for: Unique protein features of SARS-CoV-2 relative to other Sarbecoviruses
Source: Virus Evol. 2021 Jul 23;7(2):veab067. doi: 10.1093/ve/veab067 (PMC8385934; doi:10.1093/ve/veab067)
Supplement: veab067_Supp [file veab067_supp.zip › Cotten_et_al_Supplementary_materials.docx]

**Unique protein features of SARS-CoV-2 relative to other *Sarbecoviruses***

Matthew Cotten, David L. Robertson, My V.T. Phan

**Supplementary Methods**

**Generation of profile HMMs.** A set of 35 early lineage B SARS-CoV-2 were selected (see Supplementary Table 2). For each genome, all open reading frames identified, were translated and then sliced into either 44 amino acid peptides with a step size of 22 amino acids or 15 amino acid peptides with a step size of 8 amino acids. The uclust -cluster_fast algorithm with an -id setting of 0.65 from usearch [1] was used to cluster the peptides. After clustering, each cluster as aligned using MAFFT v7.477 [2] using the settings "--auto --preservecase" and each alignment was then built into a a profile HMM using HMMER-3 with hmmbuild and default settings.

**Finding pHMM matches in query genomes.** For each query genome, the matches to the pHMM libraries were sought using the HMMER-3 [3] function "hmmsearch" and setting an Eval cutoff of 0.0001. The resulting bitscores (dmn_score) for each pHMM match were retrieved from the output table. It was important to include a set of the early lineage B genomes to establish the upper limit for bitscore values to be used in the normalization. The bitscores values for all query genome were then normalized (using the x/x.max() function in Pandas v1.2 [4] for each pHMM and the entire set was then clustered and visualized using the Seaborn clustermap function [5]. The mean variance and 1-mean variance for each domain across the set of query genomes were calculated using Pandas and visualized with matplotlib [6].

**Supplementary Table 1. Close bat coronaviruses.**

| **Genome name** | **GenBank or GISAID** | **Year** | **Reference** |
| --- | --- | --- | --- |
| RpYN06 | EPI_ISL_1699446 | 2020 | GISAID [7] |
| RmYN02 | EPI_ISL_412977 | 2019 | GISAID [7] and Zhou et al. Curr Biol. 2020 Jun 8;30(11):2196-2203.e3. PMID: 32416074 |
| PrC31 | EPI_ISL_1098866 | 2018 | GISAID [7] |
| RaTG13 | MN996532 | 2013 | Zhou et al. Nature. 2020 Mar;579(7798):270-273 PMID: 32015507 |
| RshSTT182 | EPI_ISL_852604 | 2010 | GISAID (Shu and McCauley) and http://biorxiv.org/lookup/doi/10.1101/2021.01.26.428212 |
| RshSTT200 | EPI_ISL_852605 | 2010 | GISAID (Shu and McCauley) and http://biorxiv.org/lookup/doi/10.1101/2021.01.26.428212 |
| CoVZ45 | MG772933 | 2017 | Hu et al. Emerg Microbes Infect 7 (1), 154 (2018) PMID: 30209269 |
| CoVZXC21 | MG772934 | 2015 | Hu et al. Emerg Microbes Infect 7 (1), 154 (2018) PMID: 30209269 |
| RaCS203 | MW251308 | 2020 | Wacharapluesadee et al. Nat Commun. 2021 12(1):972 PMID: 33563978 |
| Rc-0319 | LC556375 | 2013 | Murakami et al. EID 2020 Dec;26(12):3025-3029 PMID: 33219796 |
| GX-P4L | MT040333 | 2017 | Lam et al. 2020. *Nature* 583:282–285. PMID: 32218527 |
| GX-P1E | MT040334 | 2017 | Lam et al. 2020. *Nature* 583:282–285. PMID: 32218527 |
| GX-P5L | MT040335 | 2017 | Lam et al. 2020. *Nature* 583:282–285. PMID: 32218527 |
| GX-P5E | MT040336 | 2017 | Lam et al. 2020. *Nature* 583:282–285. PMID: 32218527 |
| MP789 | MT121216 | 2019 | Liu et al. PLoS Pathog. 16 (5), e1008421 (2020) PMID: 32407364 |

**Supplementary Table 2. Early B lineage SARS-CoV-2 genomes used for domain libraries.**

| GenBank Accession number | Country | Collection date |
| --- | --- | --- |
| NC_045512 | China | 2019-12-15 |
| MN988668 | China | 2020-01-02 |
| MN988669 | China | 2020-01-02 |
| MN994468 | USA | 2020-01-22 |
| MN996527 | China | 2019-12-30 |
| MN996528 | China | 2019-12-30 |
| MN996529 | China | 2019-12-30 |
| MN996530 | China | 2019-12-30 |
| MN996531 | China | 2019-12-30 |
| MT007544 | Australia | 2020-01-25 |
| MT019529 | China | 2019-12-23 |
| MT019530 | China | 2019-12-30 |
| MT019531 | China | 2019-12-30 |
| MT019532 | China | 2019-12-30 |
| MT019533 | China | 2020-01-01 |
| MT020781 | Finland | 2020-01-29 |
| MT027062 | USA | 2020-01-29 |
| MT027063 | USA | 2020-01-29 |
| MT027064 | USA | 2020-01-29 |
| MT039873 | China | 2020-01-20 |
| MT039887 | USA | 2020-01-31 |
| MT039888 | USA | 2020-01-29 |
| MT039890 | South_Korea | 2020-01-01 |
| MT044258 | USA | 2020-01-27 |
| MT066176 | Taiwan | 2020-02-05 |
| MT072688 | Nepal | 2020-01-13 |
| MT093571 | Sweden | 2020-02-07 |
| MT093631 | China | 2020-01-08 |
| MT106053 | USA | 2020-02-10 |
| MT118835 | USA | 2020-02-23 |
| MT123290 | China | 2020-02-05 |
| MT123291 | China | 2020-01-29 |
| MT123292 | China | 2020-01-27 |
| MT123293 | China | 2020-01-29 |
| MT126808 | Brazil | 2020-02-28 |
| NC_045512 | China | 2019-12-15 |

**Supplementary Figure 1 to illustrate pHMM detection of amino acid changes.**

We sought to illustrate the ability of pHMMs to detect amino acid differences between a reference and a query sequence. A reference peptide containing the twenty amino acids was used to prepare a pHMM. A test set of mutant sequences was prepared by sequentially changing each amino acid to each of the other 20 amino acids. This set of 400 sequences was then queried with the wildtype 20aa profile HMM, the bit-scores describing each match were collection. The distribution of bit-scores from the 400 pHMM matches (Supplementary Figure 1a) was broad, consistent with the method's ability to report not only an amino acid changes but the type of amino acid change. The pattern of all amino acid changes across all 20 AA peptide is displayed in clustermap (Supplementary Figure 1b) with each column corresponding to a single amino acid and each row showing the score if that amino acid were changed to another amino acid. An amino acid that is frequent (e.g. alanine (A)) shows higher bit-scores across the set of changes than rarer amino acids such as cysteine (C), histidine (H), tryptophan (W) or proline (P). This spectrum closely reflects the BLOSUM62 substitution matrix [8] and demonstrates the capacity of a pHMM match both to detect changes in proteins as they evolve and to distinguish different types of changes.

**Supplementary Figure 1.** pHMM bit-score values as a measure of the type of amino acid change. A sequence encoding all 20 amino acids (ACDEFGHIKLMNPQRSTVWY) was used to prepare a pHMM. A test set of mutant sequences was prepared by changing each amino acid to each of the other 20 amino acids. This set of 400 sequences was then queried with the wildtype 20aa profile HMM, bit-scores for each match were collected in a matrix. **(a)** a histogram of all observed normalized bit-scores, the peak at 1.00 is due to changes to self (e.g. A to A change). **(b)** heatmap of normalized bit-scores, each columns represents a position in the 20 AA wt peptide, each row represents a change at that position to the indicated amino acid. The normalized bit-scores were color coded with no change from wildtype amino acid (dark grey) to the largest change from the wildtype amino acid (dark red). **(c)** Variance of normalized bit-scores from panel b were calculated for each position.

In a second analysis we examined a peptide sequence spanning the important furin cleavage site in the SARS-CoV-2 spike protein. Mutations in this region have appeared in several VOCs (A.23.1: P681R, B.1.1.7: P681H, B.1.525: Q677H) and we wanted to document the sensitivity of pHMM matching to detect single amino acid changes. Similar to Supplementary Figure 1, we prepared a pHMM from the wildtype 14aa sequence spanning the furin site. For a test set we systematically change position to each of the 20 amino acids and then gathered the bit-scores for the wildtype pHMM matching each test peptide. Similar to Supplementary Figure 1, the range of normalized bit-scores scores included a peak at 1.00 (self sequence matched to self) plus a range of lower values demonstrating the breadth of possible pHMM bits-scores for any possible single amino acid changes in the 14 amino acid peptide (Supplementary Figure 2a). The heatmap of the resulting normalized bit-scores (Supplementary Figure 2b) reveals some patterns. Most changes of the proline adjacent to the cleavage site resulted in a large reduction in bit-scores, whereas other changes resulted in detectable, distinct, but less dramatic bit-scores.

**Supplementary Figure 2. Amino acid changes across the P681 region of the spike protein.** A 14 amino acid sequence spanning the SARS-CoV-2 spike position 681 and the adjacent furin cleavage site (YQTQTNSPRRARSV) was used to prepare a pHMM. A test set of mutant sequences was prepared by changing each amino acid to each of the other 20 amino acids. This set of 280 sequences was then queried with the wildtype 14aa pHMM, bit-scores were collected. Panel a, a histogram of all observed normalized bit-scores, the peak at 1.00 due to changes to self (e.g. A to A change). Panel b, heatmap of normalized bit-scores, each columns represents a position in the 14 AA wt peptide, each row represents a change at that position to the indicated amino acid. The normalized bit-scores were color coded with no change from wildtype amino acid (dark grey) to the largest change from the wildtype amino acid (dark red). Panel C. Variance of normalized bit-scores from Panel b were calculated for each amino acid position across the peptide.

**References**

[1] R. C. Edgar, “Search and clustering orders of magnitude faster than BLAST,” *Bioinformatics*, vol. 26, no. 19, pp. 2460–2461, Oct. 2010, doi: 10.1093/bioinformatics/btq461.

[2] K. Katoh and D. M. Standley, “MAFFT Multiple Sequence Alignment Software Version 7: Improvements in Performance and Usability,” *Mol. Biol. Evol.*, vol. 30, no. 4, pp. 772–780, Apr. 2013, doi: 10.1093/molbev/mst010.

[3] S. R. Eddy, “Accelerated Profile HMM Searches,” *PLOS Comput. Biol.*, vol. 7, no. 10, p. e1002195, Oct. 2011, doi: 10.1371/journal.pcbi.1002195.

[4] Pandas, “Pandas Version 1.2”, Accessed: Jul. 06, 2021. [Online]. Available: https://pandas.pydata.org/pandas-docs/stable/index.html

[5] Seaborn, “seaborn.clustermap”, Accessed: Jul. 06, 2021. [Online]. Available: https://seaborn.pydata.org/generated/seaborn.clustermap.html

[6] matplotlib, “matplotlib”, Accessed: Jul. 06, 2021. [Online]. Available: https://matplotlib.org/

[7] Y. Shu and J. McCauley, “GISAID: Global initiative on sharing all influenza data – from vision to reality,” *Eurosurveillance*, vol. 22, no. 13, p. 30494, Mar. 2017, doi: 10.2807/1560-7917.ES.2017.22.13.30494.

[8] S. Henikoff and J. G. Henikoff, “Amino acid substitution matrices from protein blocks,” *Proc. Natl. Acad. Sci. U. S. A.*, vol. 89, no. 22, pp. 10915–10919, Nov. 1992, doi: 10.1073/pnas.89.22.10915.
